# Ocular Melanoma Metastasizing to Intra-Abdominal Lymph Nodes

**DOI:** 10.1155/2013/534730

**Published:** 2013-05-16

**Authors:** David Aranovich, Karen Meir, Michal M. Lotem, Liat Appelbaum, Hadar Merhav

**Affiliations:** ^1^Department of Surgery, Rabin Medical Center, Beilinson Hospital, 39 Jabotinsky Street, 49100 Petah Tikva, Israel; ^2^Department of Pathology, Hadassah University Hospital, Ein Kerm, P.O. Box 1200, 91120 Jerusalem, Israel; ^3^Center for Melanoma and Cancer Immunotherapy Sharett Institute of Oncology, Hadassah University Hospital, Ein Kerm, P.O. Box 1200, 91120 Jerusalem, Israel; ^4^Department of Diagnostic Radiology, Hadassah University Hospital, Ein Kerm, P.O. Box 1200, 91120 Jerusalem, Israel; ^5^Department of Surgery, Hadassah University Hospital, Ein Kerm, P.O. Box 1200, 91120 Jerusalem, Israel

## Abstract

*Background*. Visceral metastatic spread of ocular melanoma most commonly occurs via hematogenous route to the liver. Lymphatic spread of ocular melanoma into abdominal lymph nodes has not been reported previously. *Case Presentation*. A 47-year-old man with a history of ocular melanoma presented with a soft tissue mass on CT scan. The mass encased the portal structures of the hepaticoduodenal ligament. Image-guided biopsy revealed it to be a metastatic melanoma to lymph nodes. The patient underwent surgery with the intent to prolong disease-free survival. On final pathological examination, two lymph nodes were found harboring metastatic melanoma. *Conclusion*. Extrahepatic lymphatic intra-abdominal spread of ocular melanoma is not impossible. Since this mode of spread is rare, the oncologic significance of surgical resection of isolated intra-abdominal nodal with metastatic ocular melanoma is difficult to determine at the present time.

## 1. Background 

Ocular melanoma is the most frequent primary tumor of the eye. It arises from the neural crest-derived pigmented uveal epithelium of the eye in 97% of the cases, and in the remaining 3% it may appear in the conjunctiva [1]. The incidence of ocular melanoma is less than one per 100,000 inhabitants [2]. Ocular melanoma differs from the cutaneous variant not only in the mode of presentation but in the pattern of metastatic spread. While cutaneous melanoma commonly metastasizes to regional and distant lymph node basins [3], their ocular counterparts, as a rule, spread to the liver and, rarely, to other organs, such as lung, bone, skin, and central nervous system [4].

Lymphatic spread is extremely unusual for ocular melanomas. There are few case reports in the literature describing lymph node metastases of ocular melanoma to regional [5] and axillary [6] basins. To the best of our knowledge, metastatic spread of ocular melanoma to intra-abdominal lymph nodes has not been reported. 

## 2. Case Presentation

**Figure 1:**
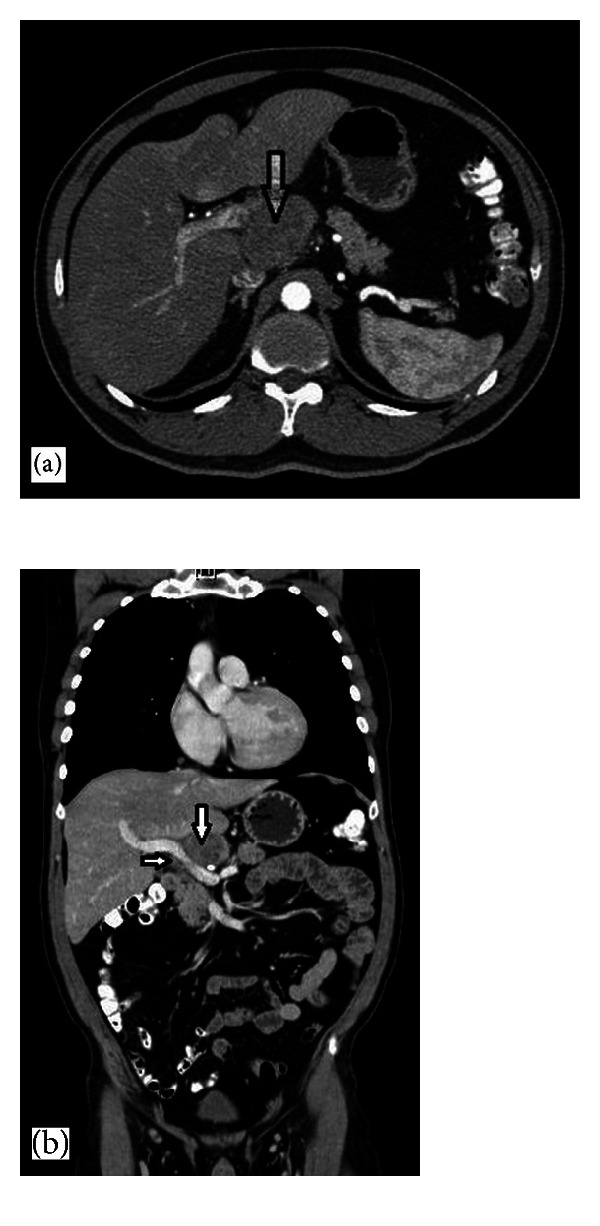
Transverse (a) and coronal (b) images of contrast enhanced CT showing upper abdominal mass located at liver hilum, containing heterogenous lymphadenopathy (arrows).

**Figure 2:**
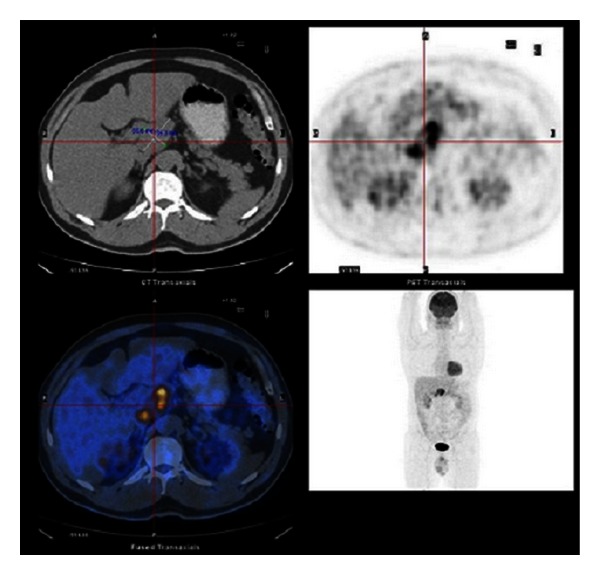
Axial and coronary images of ^18^F-FDG CT-PET. Upper and low left pictures represent plane CT and PET fusion image, respectively. Metabolically active lesion (yellow-red) located in the upper abdomen below the liver with high avidity to ^18^F-FDG on CT-PET fusion image (lower left).

**Figure 3:**
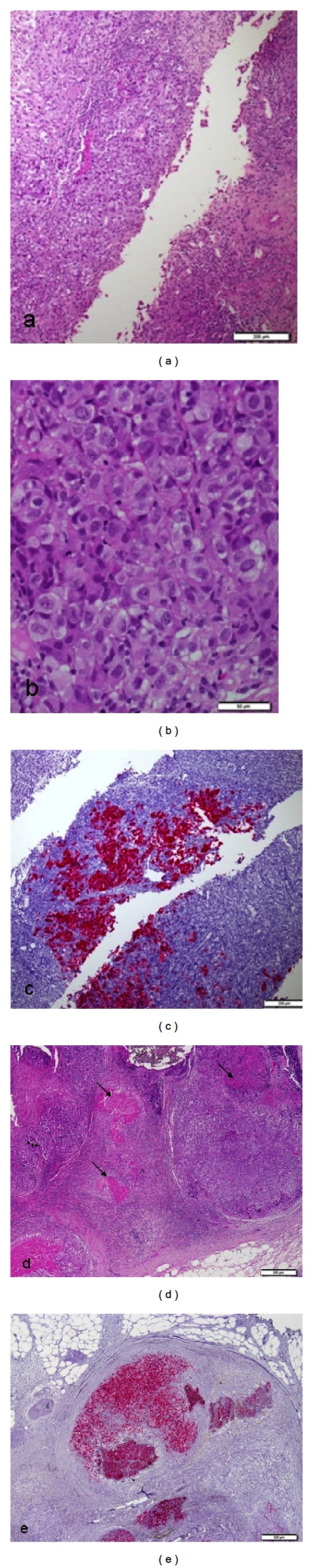
Low magnification (a) and high magnification (b) sections from the core needle biopsy showed connective tissue and a small amount of lymphoid tissue massively infiltrated by a malignant process composed of large, atypical cells with eosinophilic cytoplasm, vesicular nuclei with prominent nucleoli (hematoxylin and eosin). MART-1 immunostain confirmed the diagnosis of metastatic melanoma (c). In the resected specimen, lymph nodes matted together were almost completely replaced by metastatic melanoma with extensive areas of necrosis (arrows) ((d), low magnification, hematoxylin and eosin; (e), confirmatory MART-1 immunostain).

A 47-year-old Caucasian man presented to our center for oncological evaluation with an intra-abdominal mass, 23 months after right eye enucleation for choroidal melanoma. Physical examination was unremarkable. Full-body skin examination did not reveal any lesions of concern. Laboratory values, including liver function tests and tumour markers, were within normal limits. Computerized tomographic (CT) scan demonstrated a 5 cm abdominal mass located at the liver hilum, and displacing the body of the pancreas (Figures 1(a) and 1(b)). Positron emission tomography (PET-CT) showed marked 18-FDG avidity in the area of concern (Figure 2). A core biopsy was obtained under sonographic guidance. Histopathological examination proved it to be metastatic melanoma (Figures 3(a) and 3(b)). The patient received dimethyl triazeno imidazole carboxamide (DTIC) chemotherapy for stage IV disease [7–9]. Because of the presence of a single metastatic focus, relatively long disease free interval, and threat of mechanical interference with portal structures, should the disease progress; we favoured surgical clearance of the disease in the hope of achieving durable palliation and potentially prolonging disease-free survival (DFS). Moreover, harvesting melanoma cells would allow preparation of autologous cell vaccine to be combined with interleukin-2 therapy, to further increase the chances of successful treatment [9]. At surgery, there was a conglomerate of enlarged hard lymph nodes encasing portal structures of the hepatoduodenal ligament. The liver was normal in size and appearance. There was no gross evidence of additional metastatic disease. All grossly positive lymph nodes were surgically removed, without need of vascular inflow reconstruction or bile duct excision. Histopathologic examination of the resected specimen showed lymph nodes replaced by metastatic melanoma (Figures 3(c), 3(d), and 3(e)). The patient tolerated the procedure well, but subsequently developed severe necrotizing pancreatitis with pancreatic leak and bleeding from a hepatic artery pseudoaneurysm. He required reoperation for necrotising pancreatitis and hepatic artery angiographic stenting. He ultimately convalesced and was discharged in stable condition. He is now six months after surgery and will start adjuvant chemotherapy combined with autologous melanoma-cell vaccine. 

## 3. Discussion

Malignant melanoma is a neoplasm that develops from melanocytes. Ocular melanoma, commonly referred as uveal melanoma, may arise from any of the three parts of the uvea: choroidal melanoma, ciliary body melanoma, or iris melanoma. Melanoma of the conjunctiva is sometimes mistakenly referred to as ocular. Uveal melanoma may present with complaints of visual loss or field defect, but in many patients symptoms are lacking and the condition is discovered on routine ocular examination with a pigmented mass of the uvea [10]. Patients with metastatic melanoma have a poor prognosis, with median survival duration of only 6–9 months [11]. The etiology of ocular melanoma is unknown. Sunlight exposure, Asian descent, older age, dysplastic nevus syndrome, oculodermal melanocytosis or nevi of Ota, and increased uveal pigmentation have been implicated in the pathogenesis of ocular melanoma [12]. Tumor thickness is a major prognostic factor for the development of metastatic disease [12]. There are certain genomic abnormalities associated with poor prognosis in uveal melanoma, such as inactivation of BAP1 and loss of an entire copy of chromosome 3 (monosomy 3). Monosomy 3 strongly correlates with metastatic potential [12, 13]. Despite successful local control, the available data suggest that up to 50% of patients with intraocular melanoma will succumb to metastatic disease [12]. Unlike cutaneous melanoma, all types of ocular melanoma disseminate via the hematogenous route to the liver, and less commonly to the lung and other viscera. The entire eye and choroid have traditionally been thought of as devoid of lymphatic vessels. This concept has been challenged with discovery of lymphatic endothelium-specific markers, such as lymphatic vessel endothelial hyaluronic acid receptor (LYVE-1) and podoplanin [14]. These novel antibodies have demonstrated the presence of numerous lymphatic vessels in the conjunctiva and in the cornea [15]. It has been shown that, although, the choroid does not contain typical lymphatics; it is able to form lymphatic channels under inflammatory conditions [15]. 

Nevertheless, there are very few case reports of lymph node metastases from ocular melanoma. Dithmar et al. reported two cases of choroidal melanoma with spread to regional lymph nodes [16]. Recently, ocular melanoma metastasizing to axillary lymph nodes has been described [17]. In this present case, we report an ocular melanoma with extraocular spread to the intra-abdominal lymph node basin of the hepaticoduodenal ligament, without visceral involvement of liver, lungs, or other organs. In some tumors, surgery is an acceptable treatment for oligometastatic disease, for example, lung metastases from sarcoma, liver metastasis from colorectal, and breast cancer. In melanoma, several groups have shown promising results with resection of pulmonary [18], gastrointestinal [19], adrenal [20], and liver [21] metastases. Improved disease-free survival has been observed also with combination of chemotherapy and surgery in selected cases of metastatic uveal melanoma [21–23].

## 4. Conclusion 

Lymphatic dissemination of ocular melanoma is very rare but not impossible. Although there are no well-defined lymphatic channels in the eye to explain lymphatic spread of primary ocular malignancies, there recently is an experimental evidence that the choroid may form lymphatics under certain circumstances. The role of surgery for this condition, with or without chemo- and immunobiological therapy, is difficult to define at the present time.
